# Effect of fermented soybean meal supplementation and birth weight of broiler on performance, hematochemical parameters, and lactic acid bacteria composition under tropical conditions

**DOI:** 10.1016/j.psj.2026.107838

**Published:** 2026-09-22

**Authors:** Pawiya Pupa, Chaiyakorn Khamson, Sombat Prasongsook, Theerawit Poeikhampha, Chonticha Phumtrairat, Savek Kiatsomphob

**Affiliations:** a Department of Biology, Faculty of Science, Chulalongkorn University, Bangkok, 10330, Thailand; b Department of Animal Science, Faculty of Agriculture, Kasetsart University, Bangkok, 10900, Thailand; c Center of Excellence in Diagnosis and Monitoring of Animal Pathogens (DMAP), Faculty of Veterinary Science, Chulalongkorn University, Bangkok, 10330, Thailand; d Evershining Ingredient Co., Ltd, Samut Songkhram, 10150, Thailand

**Keywords:** Fermented soybean meal (FSBM), Broiler chicken, Lactic acid bacteria, Hematochemistry, Tropical poultry production

## Abstract

Fermented soybean meal (FSBM) is considered a potential protein source, yet its efficacy under tropical conditions requires further investigation. This study evaluated the effects of fermented soybean meal supplementation and initial body weight on growth performance, gut environment, and hematochemical parameters of broiler chickens raised under tropical conditions. A total of 1,080 male Ross 308 broilers were categorized into two hatch weight groups high (43 g) and low (33 g). Birds within each weight category were randomly assigned to diets containing 0%, 2.5%, or 5% FSBM during the starter phase (1–21 d) in a 2 × 3 factorial design. Performance data, gut health markers (lactic acid bacteria populations, cecal pH), and hematochemical profiles were analyzed. Dietary supplementation with 2.5% FSBM significantly (P < 0.05) improved overall average daily gain and feed conversion ratio compared with the control group, while the 5% inclusion level yield intermediate results. Although initial body weight significantly (P < 0.05) affected early growth performance, birds in the low weight group demonstrated compensatory growth during the finisher phase, resulting in comparable final performance between hatch weight groups. Furthermore, FSBM supplementation significantly (P < 0.05) increased lactic acid bacteria populations in both fecal and cecal samples from day 10 to day 36, accompanied by a reduction in cecal pH, indicating a more favorable intestinal environment. Fecal scores remained within acceptable ranges; however, the 5% inclusion level caused a transient increase in fecal moisture during the first week. Hematochemical parameters remained within normal physiological ranges across all treatments, indicating that FSBM supplementation did not adversely affect bird health. In conclusion, supplementation with 2.5% FSBM during the starter phase improves growth performance, enhances beneficial gut microbiota, and maintains physiological health in broilers raised under tropical conditions. These findings suggest that FSBM can be utilized as an effective nutritional strategy to improve productivity and intestinal health in tropical broiler production systems.

## Introduction

In tropical regions, the poultry industry faces substantial challenges in maintaining optimal broiler performance, largely due to high ambient temperatures and humidity, which impair growth rates and intestinal health (Kpomasse et al., 2021; Kuo et al., 2025). Although soybean meals (SBM) remains the primary protein source in broiler diets (Olukosi et al., 2019; Anjos et al., 2025), its utilization is constrained by the presence of anti-nutritional factors (ANFs), such as trypsin inhibitors and phytates. These compounds interfere with nutrient absorption and trigger inflammatory responses within the developing gut (Salim et al., 2023; David et al., 2024).

Fermentation has emerged as a robust processing technology for enhancing the nutritional profile of soybean meals. Co-fermentation with bacteria (e.g., *Lactobacillus* or *Bacillus*) effectively reduces anti nutritional factors (ANFs) while facilitating the hydrolysis of complex proteins into smaller, highly digestible bioactive peptides (Madibana et al., 2022; Mulalapele et al., 2026). Furthermore, fermented soybean meal (FSBM) serves as a functional feed ingredient that modulates the gut microbiota by fostering the proliferation of beneficial lactic acid bacteria (LAB) (Li et al., 2020) and promoting the synthesis of organic acids, which subsequently reduce intestinal pH (Sugiharto and Ranjitkar, 2019). These physiological modifications create a protective environment against pathogenic colonization and enhance overall feed efficiency (Chen et al., 2021).

Another critical factor influencing the efficiency of broiler production is the variability in initial hatch weight (Souza da Silva et al., 2021). Such disparities often result in flock heterogeneity, with low weight chicks frequently encountering challenges in resource competition during the early rearing period (Tona et al., 2004). While lower weight chicks typically possess a developmental disadvantage, they retain the physiological potential for compensatory growth during later production phases, provided that they are supported by optimal nutritional strategies to stabilize their intestinal microenvironment (Zhan et al., 2007). However, in tropical production systems, this growth trajectory is frequently hindered by high ambient temperatures and humidity, which exacerbates metabolic stress and compromise intestinal integrity (Goo et al., 2019). Consequently, achieving compensatory growth under such challenging conditions requires targeted nutritional interventions. FSBM supplementation may enable lower weight chicks to overcome initial developmental limitations and attain performance levels comparable to their larger counterparts, even within the constraints of tropical environments (Zhao et al., 2025; Mulalapele et al., 2026).

While the benefits of FSBM are well-documented, data regarding the interaction between FSBM supplementation and initial hatch weight, particularly under the environmental stressors of tropical open-sided housing, remains limited. Accordingly, this study aimed to evaluate the effects of replacing standard soybean meal with FSBM at varying inclusion levels on the growth performance, hematochemical parameters, and gut microbial composition in broiler chickens stratified by hatch weight. The findings will establish whether FSBM serves as a robust nutritional strategy to optimize performance and promote flock uniformity within tropical broiler production systems.

## Materials and methods

### Ethical approval and animal care

The experimental procedures were approved by the Institutional Animal Care and Use Committee of Kasetsart University, Bangkok, Thailand (Protocol number: ACKU67-AGR-003). Animal handling and management practices were conducted in accordance with the principles and guidelines established in the *Guide for the Care and Use of Agricultural Animals in Research and Teaching* (4th edition, 2020). Euthanasia procedures adhered to the *American Veterinary Medical Association (AVMA) Guidelines for the Euthanasia of Animals* (2020 Edition). Birds were individually weighed and euthanized using CO_2_ asphyxiation.

### Preparation of FSBM

A commercial FSBM product (ESP 500, Lot. 0236; Evershining Ingredient Co., Ltd., Samut Songkhram, Thailand) was used in this study. The analyzed nutrient composition of the product was 50.39% crude protein, 0.53% crude fat, 3.25% crude fiber, 6.61% ash, and 72.26% KOH protein solubility. According to the manufacturer, the FSBM was prepared via fermentation using a pure culture of *Lactobacillus acidophilus*. The product was directly incorporated into the experimental diets.

### Animal, experimental design, and husbandry

The study was conducted at the Animal Research Farm, Department of Animal Science, Faculty of Agriculture, Kasetsart University, Bangkok, Thailand. A total of 1,080 male broiler chickens (ROSS 308) were utilized in this experiment. The birds were reared in an open-sided house for 39 days. The facility was divided into 36 floor pens (1.5 × 3.0 m), with concrete flooring covered with rice husk litter to a depth of 5 cm. Environmental conditions were monitored throughout the study, with an average temperature of 31.14 ± 1.12°C and an average relative humidity of 77.59 ± 2.89%. All birds were vaccinated against Newcastle disease and infectious bronchitis on the day of hatch and again at 10 days of age, following standard vaccination protocols.

Birds were randomly allocated to six experimental treatments in a 2 × 3 factorial arrangement within a completely randomized design, with six replicates per treatment and 30 birds per pen. The first factor was initial hatch weight, where chicks were stratified into two groups: high hatch weight (high; 43 ± 0.09 g) and low hatch weight (low; 33 ± 0.06 g). The second factor was the dietary supplementation of FSBM at three inclusion levels: 0%, 2.5%, and 5%. Experimental diets were formulated for three feeding phases: starter (1–21 d), grower (22–32 d), and finisher (33–39 d). Birds were assigned to one of three dietary treatments: a basal diet (control), the basal diet supplemented with 2.5% FSBM (FSBM2.5), or the basal diet supplemented with 5% FSBM (FSBM5), following the protocol described by Khamson, et al. (2025). FSBM was included in the diet solely during the starter phase. During the grower and finisher phases, all birds were fed the basal diet without FSBM supplementation. All experimental diets were manufactured by Top Feed Mill Co., Ltd., Pathum Thani, Thailand. The ingredient composition and nutrient content of the diets are presented in Table 1, Table 2.

**Table 1:** Composition of experimental diets in this study.

Ingredient *(*%*)*	Experimental diets^a^		
	0%	2.5%	5%
Corn	45.27	45.17	45.07
Wheat	8.00	8.00	8.00
Palm oil	1.30	1.30	1.30
Rice solvent bran	8.00	8.00	8.00
Soybean 48%	26.69	24.11	21.52
Full fat soybean meal	4.00	4.00	4.00
Fermented soybean meal	0.00	2.50	5.00
Pork meal 50%	3.00	3.00	3.00
L-Lysine	0.45	0.45	0.46
DL-Methionine	0.43	0.43	0.43
L-Threonine	0.19	0.19	0.18
MCP-21^b^	0.42	0.42	0.43
Calcium carbonate	1.48	1.66	1.84
Sodium chloride	0.30	0.30	0.30
Enzymes^c^	0.01	0.01	0.01
Choline Chloride 60%	0.28	0.28	0.28
Vitamin & mineral premix^d^	0.18	0.18	0.18
Total	100.00	100.00	100.00

a Diet with different level of fermented soybean meal (FSBM) supplementation.

b MCP-21 = 12% Monocalcium phosphate.

c Phytase was supplemented at 1,000 FTU/kg of diet. The NSP-degrading enzyme, containing endo-1,4-β-xylanase and endo-1,4-β-glucanase, was supplemented at 560 TXU and 250 TGU/kg of diet, respectively, according to the manufacturer’s recommended dosage.

d Vitamin and mineral premix was formulated to meet the Ross 308 broiler recommendations and provided the following added nutrients per kilogram of complete starter diet: vitamin A, 13,000 IU; vitamin D₃, 5,000 IU; vitamin E, 80 IU; vitamin K (menadione), 4.0 mg; thiamin (B₁), 5 mg; riboflavin (B₂), 9 mg; niacin, 70 mg; pantothenic acid, 25 mg; pyridoxine (B₆), 5 mg; biotin, 0.35 mg; folic acid, 2.5 mg; vitamin B₁₂, 0.02 mg; Cu, 16 mg; I, 1.25 mg; Fe, 20 mg; Mn, 120 mg; Se, 0.30 mg; and Zn, 120 mg.

**Table 2:** Nutritional value of diets in this study.

Nutritional value	Experimental diets^a^		
	0%	2.5%	5%
ME.^b^ For Poultry, Kcal/kg	2975	2975	2975
Protein, %	23.00	23.00	23.00
Fat, %	4.34	4.37	4.40
Fiber, %	4.10	4.03	3.96
Calcium, %	1.23	1.30	1.37
Total P, %	0.89	0.89	0.89
Availble P, %	0.50	0.50	0.50
Sodium, %	0.12	0.12	0.12
Chloride, %	0.23	0.23	0.23
Lysine, %	1.51	1.51	1.50
Methionine + Cystine, %	1.08	1.08	1.08
Methionine, %	0.75	0.75	0.76
Threonine, %	1.03	1.02	1.02
Tryptophan, %	0.26	0.26	0.26
Valine, %	1.04	1.05	1.07
Choline, mg	1700.00	1700.00	1700.00

a Diet with different level of fermented soybean meal (FSBM) supplementation.

b ME. = Metabolized energy.

### Growth performance evaluation

Broilers were weighed by pen on days 1, 21, 32, and 39, which correspond to the initial placement and the conclusion of the starter, grower, and finisher feeding phases, respectively. Feed intake was determined by calculating the difference between the weight of feed provided and the weight of residual feed at the end of each period. Performance metrics, including average daily gain (ADG), average daily feed intake (ADFI), and feed conversion ratio (FCR), were calculated for the starter (1–21 d), grower (22–32 d), and finisher (33–39 d) phases. General flock health was monitored daily throughout the experiment. The incidence of illness or mortality was recorded to calculate the morbidity and mortality rates for each experimental group (Kim et al., 2017; Zhao et al., 2025).

### Fecal lactic acid bacteria count

The fecal samples were randomly collected from one bird per replicate on days 1,

10, 20, 30, and 36. Samples were aseptically obtained via cloacal swab, transferred into the sterile plastic tube, placed on ice, and transported immediately to the laboratory for analysis. To enumerate viable LAB, fecal samples from one representative bird per group were homogenized with sterile normal saline at a 1:9 (w/v) ratio. The fecal suspensions were serially diluted and plated onto MRS agar (Becton, Dickinson and Company, Maryland, USA) using the spread plate method. Plates were then incubated at 37°C for 48 h. Microbial counts were determined in triplicate and calculated as log colony forming units (CFU) per g (Pupa et al., 2021).

### Fecal score determination

Fecal scores were assessed using samples from the same representative birds selected for the microbial count, employing a five-point scoring system (Feng et al., 2024). Scores were determined by three professionals observers who were blinded to the treatment assignments. The scoring criteria were defined as follows: a score of 4 indicated very watery stool lacking any defined shape; score 3 denoted very watery, shapeless feces with a significant portion of the surface covered by feed granules; score 2 described watery, shapeless stools; score 1 corresponded to soft feces retaining a mostly complete columnar shape; score 0 represented dry feces maintaining a fully formed columnar shape, indicating normal conditions.

### Hematological and biochemical parameters analysis

Blood samples were collected from one bird per replicate on day 20. Approximately 4 mL of blood were drawn via venipuncture of the left brachial (wing) vein. Two milliliters were transferred into an EDTA-coated tube for complete blood count (CBC) analysis, and the remaining 2 mL were transferred into a heparinized tube for biochemical profiling. The CBC, including red blood cell (RBC) count, hemoglobin (HGB), hematocrit (HCT), mean corpuscular volume (MCV), mean corpuscular hemoglobin concentration (MCHC), thrombocytes, and white blood cell (WBC) count, was performed using an automated hematology analyzer (ADVIA 2120i; Bayer HealthCare, NJ, USA), combining with manually differential leukocyte counts (heterophils, lymphocytes, monocytes, eosinophils, and basophils) were determined manually via blood smear examination (Natt and Herrick, 1952). Biochemical parameters, including aspartate aminotransferase (AST), alanine aminotransferase (ALT), creatine kinase (CK), blood urea nitrogen (BUN), total protein, albumin, globulin, triglycerides, and cholesterol, were determined using an automated clinical chemistry analyzer (Mindray BS-800 M Chemistry Analyzer; Mindray Bio-Medical Electronics, Shenzhen, China) (Im et al., 2025).

### Cecal lactic acid bacteria count and pH determination

The cecal content was aseptically collected from one randomly selected bird per replicate following euthanasia on days 20 and 36. Samples were placed in sterile plastic tubes, kept on ice, and transported immediately to the laboratory for analysis. For LAB enumeration, cecal content samples were subjected to 10-fold serial dilution in sterile normal saline. Appropriate dilutions were plated onto MRS agar (Becton, Dickinson and Company, MD, USA). The plates were incubated at 37°C for 48 h under anaerobic conditions using the candle jar technique. Microbial counts were determined in triplicate and expressed as log CFU/g (Qaid et al., 2021). The pH of the cecal content was measured in triplicate by mixing 1 g of the sample with 2 mL of distilled water, followed by analysis using a pH meter (Hanna HI98100; Hanna Instruments, Inc., RI, USA) according to the manufacturer’s protocol (Soumeh et al., 2019). Additionally, feed samples from each experimental group were collected prior to distribution. To determine the feed pH, 2 g of the sample was diluted with 18 mL of distilled water and analyzed following the same procedure as used for the cecal samples (Shi et al., 2017).

### Statistical analysis

All statistical analyses were performed using GraphPad Prism version 10 for macOS (GraphPad Software, San Diego, CA, USA). Results for normally distributed data are presented as mean ± standard error of the mean (SEM). Data were analyzed as a 2 × 3 factorial arrangement in a completely randomized design. The main effects of initial hatch weight, dietary FSBM level, and their interaction were evaluated using analysis of variance (ANOVA). When significant effects were detected, mean differences among treatments were separated using Tukey’s multiple comparison test. Statistical significance was declared at P < 0.05.

## Results and discussion

### Growth performance

The effects of FSBM supplementation level and initial hatch weight (chick weight: high and low) on ADFI are presented in Table 3. Across the growth phases, no significant interaction was observed between supplementation level and bird hatch size. During the starter phase, FSBM supplementation did not affect ADFI; however, bird weight significantly influenced feed consumption, with high weight birds consuming more feed (55.71 ± 0.25 g) compared to low weight birds (54.30 ± 0.24 g). In the grower phase, both supplementation level and bird hatch weight significantly influenced ADFI as main effects. Birds in the control group exhibited significantly higher ADFI (132.38 ± 2.28 g) than those in FSBM2.5 and FSBM5 groups (126.46 ± 3.03 g and 127.45 ± 2.18 g, respectively). Consistent with the starter phase, high weight birds maintained a higher ADFI than low weight birds (131.26 ± 1.70 g and 126.27 ± 1.99 g, respectively). Conversely, the effect of initial hatch weight reversed during the finisher phase; low weight birds consumed significantly more feed (149.22 ± 1.78 g) than high weight birds (141.82 ± 0.85 g). Over the entire experimental duration, no significant differences in ADFI were observed regarding FSBM supplementation or bird weight.

**Table 3:** Means (± SEM) for bird initial hatch weight and supplementation level of fermented soybean meal on average daily feed intake of chick raised to 39 days.

Average daily feed intake (g)						
Supplementation level	Bird initial hatch weight			P-value		
	High	Low	Mean	S	B	S*B
Starter (1-21 d)						
CON	56.20 ± 0.99	54.76 ± 2.03	55.48 ± 0.73	0.8805	0.0152^ψ^	0.9993
FSBM2.5	55.41 ± 1.07	53.96 ± 1.54	54.69 ± 0.73			
FSBM5	55.51 ± 0.59	54.18 ± 2.23	54.84 ± 0.67			
Mean	55.71 ± 0.25^Y^	54.30 ± 0.24^X^				
Grower (21-32 d)						
CON	134.66 ± 2.61	130.10 ± 2.99	132.38 ± 2.28^B^	0.0170^ψ^	0.0477^ψ^	0.9215
FSBM2.5	129.48 ± 1.84	123.43 ± 1.43	126.46 ± 3.03^A^			
FSBM5	129.63 ± 2.60	125.27 ± 1.93	127.45 ± 2.18^A^			
Mean	131.26 ± 1.70^Y^	126.27 ± 1.99^X^				
Finisher (32-39 d)						
CON	143.51 ± 6.74	152.30 ± 5.06	147.91 ± 4.40	0.5460	0.0200^ψ^	0.8959
FSBM2.5	140.86 ± 2.66	146.12 ± 3.36	143.49 ± 2.63			
FSBM5	141.09 ± 6.33	149.23 ± 2.07	145.16 ± 4.07			
Mean	141.82 ± 0.85^X^	149.22 ± 1.78^Y^				
Overall (1-39 d)						
CON	100.55 ± 2.41	100.76 ± 1.44	100.66 ± 0.10	0.5605	0.2221	0.7514
FSBM2.5	98.62 ± 1.04	98.21 ± 0.54	98.42 ± 0.21			
FSBM5	99.59 ± 2.21	97.12 ± 1.46	98.36 ± 1.23			
Mean	99.59 ± 0.56	98.70 ± 1.08				
^abc/xy^ Means with different superscript within a column (abc) or row (xy) differ significantly. ^ABC/XY^ Means with different superscript within a column (ABC) or row (XY) differ significantly.						
^ψ^ Significant effects of supplementation level feed group (S), bird weight (B) or their interaction (S*B)						

The results indicate that while initial hatch weight significantly influenced feeding behavior across different growth stages, FSBM supplementation primarily affected feed intake during the grower phase. These findings contrast with previous studies, which typically reported that FSBM supplementation does not significantly influence feed consumption (Li et al., 2020; Husnain et al., 2025; Obeidat et al., 2025). The significant reduction in ADFI during the grower phase (21–32 d) for birds fed FSBM (both 2.5% and 5.0%) suggests that fermentation may have altered either the nutrient density or the palatability of the diet. Typically, fermentation increases the bioavailability of amino acids and reduces ANFs (Li et al., 2022; Shi et al., 2017), which can lead to improved feed efficiency (Chen et al., 2021). If the birds’ metabolic energy requirements are satisfied more efficiently due to higher nutrient digestibility of FSBM, they may naturally down-regulate voluntary intake to maintain energy homeostasis (Ferket and Gernat, 2006). Alternatively, the decline in feed intake may be attributed to shifts in the feed’s organoleptic profile. Specifically, the presence of fermentation-derived metabolites, such as organic acids, may modify the sensory profile of the diet (Canibe and Jensen, 2012; Missotten et al., 2015), necessitating an acclimatization period that leads to a temporary reduction in feed consumption (Predescu et al., 2024). The fact that this decline in ADFI did not persist through the finisher phase or the overall experimental period suggests a gradual adaptation of the gastrointestinal tract to the fermented substrate over time (Canibe and Jensen, 2012; Predescu et al., 2024). This recovery in feed consumption during the final stage indicates that the birds had achieved full physiological adaptation to the dietary organic acids and fermentation metabolites, thereby stabilizing their intake once the initial acclimatization period was surpassed. The shift in intake patterns between high and low weight birds is noteworthy. In the early stages (starter and grower), high weight birds consumed more feed, consistent with the higher metabolic demands required to maintain their greater body mass (Lasiewski and Dawson, 1967; Yahaya et al., 2012). However, the reversal observed in the finisher phase—where low weight birds exhibited higher relative feed intake—may indicate a pattern of growth recovery. This dynamic reflects the birds’ innate metabolic drive to optimize development, wherein low weight bird individuals actively adjust their intake to accelerate growth and align with the standard growth trajectory (Leeson and Summer, 2009). This growth recovery pattern suggests that even under challenging tropical conditions, where high ambient temperature and humidity often compromise broiler growth and intestinal health, FSBM supplementation potentially enables low weight birds to achieve compensatory growth.

The effects of FSBM supplementation and bird weight on ADG are summarized in Table 4. No significant interaction was observed between FSBM supplementation and initial hatch weight across the growth phases. During the starter phase, supplementation levels did not significantly influence ADG. In contrast, initial hatch weight had a highly significant impact on weight gain, with high weight birds achieving a higher ADG (48.50 ± 0.08 g) compared to low weight birds (43.55 ± 0.18 g). In the grower phase, FSBM supplementation significantly influenced ADG. Birds in the FSBM2.5 group showed the highest growth rate (81.99 ± 0.52 g), which was significantly greater than the control group (78.06 ± 0.81 g), while the FSBM5 group (79.17 ± 0.46 g) did not differ significantly from either the control or the FSBM2.5 group. During the finisher phase, supplementation levels did not significantly affect ADG; however, initial hatch weight exerted a significant effect, with low weight birds exhibiting a higher ADG (81.69 ± 1.47 g) compared to high weight birds (69.46 ± 2.31 g). Over the total experimental period, FSBM supplementation significantly impacted overall ADG. Specifically, the FSBM2.5 group achieved a higher overall ADG (66.07 ± 0.36 g) than both the control (64.01 ± 0.19 g) and FSBM5 (64.38 ± 0.17 g) groups.

**Table 4:** Means (± SEM) for bird initial hatch weight and supplementation level of fermented soybean meal on average daily gain of chick raised to 39 days.

Average daily gain (g)						
Supplementation level	Bird initial hatch weight			P-value		
	High	Low	Mean	S	B	S*B
Starter (1-21 d)						
CON	48.36 ± 0.32^y^	43.23 ± 0.33^x^	45.79 ± 2.57	0.6565	< 0.0001^ψ^	0.9315
FSBM2.5	48.62 ± 0.93^y^	43.86 ± 0.44^x^	46.24 ± 2.39			
FSBM5	48.51 ± 0.33^y^	43.56. ± 0.38^x^	46.03 ± 2.48			
Mean	48.50 ± 0.08^Y^	43.55 ± 0.18^X^				
Grower (21-32 d)						
CON	77.25 ± 2.36	78.87 ± 0.76	78.06 ± 0.81^A^	0.0424^ψ^	0.6145	0.2132
FSBM2.5	82.51 ± 3.19	81.47 ± 1.59	81.99 ± 0.52^B^			
FSBM5	78.71 ± 0.50	79.62 ± 1.92	79.17 ± 0.46^AB^			
Mean	79.49 ± 1.57	79.99 ± 0.77				
Finisher (32-39 d)						
CON	65.00 ± 4.27^x^	80.08 ± 5.70^y^	72.54 ± 7.54	0.1478	0.0333^ψ^	0.6737
FSBM2.5	72.75 ± 5.09	84.62 ± 3.33	78.69 ± 5.94			
FSBM5	70.61 ± 3.47	80.35 ± 3.13	75.48 ± 4.87			
Mean	69.46 ± 2.31^X^	81.69 ± 1.47^Y^				
Overall (1-39 d)						
CON	63.82 ± 0.82	64.20 ± 0.83	64.01 ± 0.19^A^	0.0208^ψ^	0.9982	0.8965
FSBM2.5	66.23 ± 2.09	65.70 ± 0.75	66.07 ± 0.36^B^			
FSBM5	64.21 ± 1.88	64.54 ± 1.56	64.38 ± 0.17^A^			
Mean	64.82 ± 0.81	64.82 ± 0.45				
^abc/xy^ Means with different superscript within a column (abc) or row (xy) differ significantly. ^ABC/XY^ Means with different superscript within a column (ABC) or row (XY) differ significantly.						
^ψ^ Significant effects of supplementation level feed group (S), bird weight (B) or their interaction (S*B)						

The significant increase in ADG during the grower phase and the overall period in the FSBM2.5 group suggests FSBM supplementation, particularly at a 2.5% inclusion level, exerts a notable growth-promoting effect. These findings are consistent with previous reports of improved ADG during the grower phase and the overall period in broilers supplemented with 5% or 7.5% FSBM (Zhao et al., 2025), as well as in birds fed diets where 25% of SBM was supplemented with FSBM (Li et al., 2020). Furthermore, these results align with the study of Husnain et al. (2025), who reported that 2.5% FSBM supplementation significantly enhanced weight gain in broilers during the starter and grower phases, as well as throughout the entire experimental period. The observed increase in ADG within the FSBM-supplemented groups indicates that the inclusion of fermented soybean meal enhances the overall nutritional value of the diet, thereby promoting superior growth performance. It is well-established that the fermentation process effectively reduces the concentration of ANFs, such as trypsin inhibitors and phytates, while simultaneously hydrolyzing large protein molecules into smaller, highly bioavailable peptides (Feng et al., 2007; Kim et al., 2016). These low-molecular-weight peptides are more readily absorbed by the developing intestinal tract of broilers, allowing for more efficient nutrient partitioning toward muscle deposition and growth (Shi et al., 2017; Wang et al., 2024). Interestingly, the superior performance of the 2.5% inclusion level compared to 5% suggests an optimal supplementation threshold, which aligns with prior findings reporting that lower inclusion levels may yield more favorable outcomes (Li et al., 2020; Husnain et al., 2025; Zhao et al., 2025). This effect may be attributed to the possibility that the higher inclusion levels introduce dietary imbalances or create a sensory overload—potentially driven by higher concentrations of fermentation-derived metabolites—that may outweigh the associated nutritional benefits (Alabi and Adedokun, 2025). This aligns with observations of a decreasing trend in body weight when higher levels of FSBM are supplemented (Irawan et al., 2022). Regarding initial hatch weight, the starter phase results confirmed that high weight chicks naturally grow faster initially due to higher metabolic momentum (Tona et al., 2004). However, the absence of significant weight differences in the grower phase, followed by a reversal in the finisher phase, where low weight birds outperformed high weight birds, strongly supports our hypothesis regarding the growth recovery pattern. This is evidenced by the identical overall mean ADG observed in both initial hatch weight groups, indicating that initial hatch size variation did not dictate final growth performance.

The effects of FSBM supplementation and initial hatch weight on FCR are detailed in Table 5. No significant interaction was observed between FSBM supplementation and initial hatch weight across the growth phases. During the starter phase, FCR was affected only by initial hatch weight; high weight birds exhibited a significantly lower FCR (1.15 ± 0.01) compared to low weight birds (1.25 ± 0.01). In contrast, during the grower phase, no significant effect of initial hatch weight was observed; however, both the FSBM2.5 (1.55 ± 0.03) and FSBM5 (1.61 ± 0.03) groups achieved significantly lower FCR values compared to the control group (1.70 ± 0.05). During the finisher period, supplementation did not influence FCR; however, low weight birds demonstrated superior feed efficiency (1.85 ± 0.06) compared to high weight birds (2.08 ± 0.06). For the total duration of the study, birds in the FSBM2.5 group showed the most efficient FCR (1.49 ± 0.01), which was significantly lower than that of the control group (1.57 ± 0.01), whereas the FCR of the FSBM5 group (1.53 ± 0.02) was intermediate and did not differ significantly from the other two groups.

**Table 5:** Means (± SEM) for bird initial hatch weight and supplementation level of fermented soybean meal on feed conversion ratio of chick raised to 39 days.

Feed conversion ratio						
Supplementation level	Bird initial hatch weight			P-value		
	High	Low	Mean	S	B	S*B
Starter (1-21 d)						
CON	1.16 ± 0.02^x^	1.27 ± 0.05^y^	1.22 ± 0.05	0.7349	0.0016^ψ^	0.9783
FSBM2.5	1.14 ± 0.03	1.23 ± 0.03	1.19 ± 0.05			
FSBM5	1.15 ± 0.02^x^	1.24 ± 0.04^y^	1.19 ± 0.05			
Mean	1.15 ± 0.01^X^	1.25 ± 0.01^Y^				
Grower (21-32 d)						
CON	1.75 ± 0.05	1.65 ± 0.05	1.70 ± 0.05^B^	0.0331^ψ^	0.0808	0.9348
FSBM2.5	1.58 ± 0.07	1.52 ± 0.04	1.55 ± 0.03^A^			
FSBM5	1.65 ± 0.03	1.58 ± 0.06	1.61 ± 0.03^A^			
Mean	1.66 ± 0.05	1.58 ± 0.04				
Finisher (32-39 d)						
CON	2.19 ± 0.13	1.93 ± 0.09	2.06 ± 0.13	0.2637	0.0487^ψ^	0.9722
FSBM2.5	1.98 ± 0.13	1.74 ± 0.06	1.86 ± 0.12			
FSBM5	2.07 ± 0.16	1.87 ± 0.07	1.97 ± 0.10			
Mean	2.08 ± 0.06^Y^	1.85 ± 0.06^X^				
Overall (1-39 d)						
CON	1.58 ± 0.03	1.57 ± 0.03	1.57 ± 0.01^B^	0.0464^ψ^	0.4553	0.8247
FSBM2.5	1.49 ± 0.05	1.50 ± 0.02	1.49 ± 0.01^A^			
FSBM5	1.55 ± 0.02	1.51 ± 0.05	1.53 ± 0.02^AB^			
Mean	1.54 ± 0.03	1.53 ± 0.02				
^abc/xy^ Means with different superscript within a column (abc) or row (xy) differ significantly. ^ABC/XY^ Means with different superscript within a column (ABC) or row (XY) differ significantly.						
^ψ^ Significant effects of supplementation level feed group (S), bird weight (B) or their interaction (S*B)						

The significant improvement in FCR observed during the grower phase and the overall experimental period in the FSBM2.5 group suggests that supplementation at this level enhances feed conversion efficiency throughout the production cycle. These findings are consistent with Husnain et al. (2025), who reported that birds fed 2.5%, 5%, and 7.5% FSBM exhibited improved FCR during the starter and grower phases, as well as the overall period. Similarly, birds fed diets in which 25% and 50% of soybean meal was replaced with FSBM showed improved FCR during the grower phase and the overall period, aligning with Zhao et al. (2025), who demonstrated enhanced feed efficiency in birds supplemented with 5% FSBM. Conversely, these findings contrast with several studies that did not demonstrate any significant effect of FSBM on FCR (Guo et al., 2020; Obeidat et al., 2025). The ability of 2.5% FSBM supplementation to enhance FCR indicates that FSBM facilitates superior dietary nutrient utilization. This improvement is further evidenced by the observed increase in ADG despite a reduction in ADFI during the grower phase, highlighting a more efficient metabolic response to the fermented supplementary diet. By reducing ANFs and providing bioactive peptides and organic acids, FSBM likely improves the morphology of the intestinal villi and the balance of the cecal microbiota (Li et al., 2020; Obeidat et al., 2025). Such physiological enhancements allow the birds to extract and assimilate nutrients more effectively from a smaller volume of feed, resulting in a more favorable (lower) FCR (Yi et al., 2024; Zhao et al., 2025). The fact that 2.5% inclusion level yielded the most efficient results suggests that this concentration provides the optimal balance for gastrointestinal health and metabolic demand. Furthermore, the FCR data reveals a distinct shift in efficiency relative to initial hatch size. During the starter phase, high weight birds were more efficient, likely due to better-developed metabolic systems at hatching (Tona et al., 2004). However, in the finisher phase, the low weight birds exhibited significantly higher efficiency. This shift also supports the concept of growth recovery pattern (Leeson and Summer, 2009). Although low weight birds consumed more feed during the finisher phase, they converted that intake into body mass more effectively than their larger counterparts. This may be attributed to the fact that lower weight chicks possess a higher potential for efficient late-stage growth once their digestive and metabolic systems reach functional maturity (Khulel and Sabri, 2020; Yang et al., 1996).

Notably, the absence of a significant interaction between supplementation level and initial hatch weight across all growth phases for any of the growth performance parameters indicates that the response to FSBM supplementation is consistent, irrespective of initial hatch size. This is a significant finding for the poultry industry, suggesting that FSBM can be effectively implemented as a nutritional strategy in heterogeneous flocks without disparate effects on performance across different weight classes. Based on the significant improvements in ADG and FCR, this study recommends a 2.5% inclusion rate of FSBM as a viable nutritional strategy for broiler production. This approach not only optimizes biological performance but also promotes flock uniformity by supporting the growth recovery potential of lower weight birds within the population.

### Lactic acid bacterial count and pH value

The effects of FSBM supplementation level and initial hatch weight on the fecal and cecal LAB count are presented in Table 6, Table 7, respectively. No significant interactions were observed between supplementation level and initial hatch weight for either fecal or cecal LAB populations. At day 1, only initial hatch weight significantly influenced the fecal LAB count, with low weight birds harboring a higher initial density (5.35 ± 0.20 log CFU/g) compared to high weight birds (3.94 ± 0.38 log CFU/g). By day 10, FSBM supplementation became the dominant factor; both FSBM2.5 and FSBM5 groups showed significantly higher LAB counts (8.70 ± 0.17 and 8.65 ± 0.02 log CFU/g, respectively) than the control group (7.83 ± 0.08 log CFU/g). At day 20, both supplementation level and initial hatch weight independently affected the fecal population. The FSBM2.5 group achieved the highest LAB density (9.32 ± 0.11 log CFU/g), while high weight birds (9.25 ± 0.17 log CFU/g) also maintained a significantly higher count compared to low weight birds (8.87 ± 0.23 log CFU/g). By days 30 and 36, the influence of FSBM supplementation remained highly significant, with birds receiving either 2.5% or 5% FSBM maintaining superior LAB levels compared to the control. The microbial patterns in the cecal contents mirrored those observed in the feces. At day 20, both FSBM supplementation and initial hatch weight significantly influenced cecal LAB counts. Birds receiving 2.5% and 5% FSBM exhibited significantly higher LAB populations (9.19 ± 0.14, 8.97 ± 0.17 log CFU/g, respectively) than the control group (8.53 ± 0.01 log CFU/g). Similar to the fecal results, high weight birds maintained a significantly higher cecal LAB count (9.00 ± 0.24 log CFU/g) than low weight birds (8.79 ± 0.15 log CFU/g). By day 36, only the effect of FSBM supplementation remained highly significant, with both the FSBM2.5 (8.88 ± 0.04 log CFU/g) and FSBM5 (8.63 ± 0.07 log CFU/g) groups sustaining significantly higher LAB density compared to the control group (8.17 ± 0.03 log CFU/g).

**Table 6:** Means (± SEM) for bird initial hatch weight and supplementation level of fermented soybean meal on lactic acid bacterial count from feces of chick raised to 39 days.

Fecal lactic acid bacterial count (log CFU/g)						
Supplementation level	Bird initial hatch weight			P-value		
	High	Low	Mean	S	B	S*B
Day 1						
CON	3.28 ± 0.60	4.96 ± 0.11	4.12 ± 0.84	0.2698	0.0326^ψ^	0.8427
FSBM2.5	3.96 ± 0.29	5.48 ± 0.32	4.72 ± 0.76			
FSBM5	4.58 ± 0.94	5.61 ± 0.82	5.09 ± 0.52			
Mean	3.94 ± 0.38^X^	5.35 ± 0.20^Y^				
Day 10						
CON	7.91 ± 0.12^a^	7.74 ± 0.27^a^	7.83 ± 0.08^A^	< 0.001^ψ^	0.6145	0.2132
FSBM2.5	8.52 ± 0.08^b^	8.87 ± 0.06^b^	8.70 ± 0.17^B^			
FSBM5	8.68 ± 0.07^b^	8.63 ± 0.04^b^	8.65 ± 0.02^B^			
Mean	8.37 ± 0.23	8.41 ± 0.34				
Day 20						
CON	8.90 ± 0.09^ay^	8.44 ± 0.17^ax^	8.67 ± 0.23^A^	< 0.001^ψ^	0.0162^ψ^	0.3628
FSBM2.5	9.42 ± 0.08^b^	9.21 ± 0.03^b^	9.32 ± 0.11^B^			
FSBM5	9.41 ± 0.07^by^	8.96 ± 0.15^bx^	9.18 ± 0.23^B^			
Mean	9.25 ± 0.17^Y^	8.87 ± 0.23^X^				
Day 30						
CON	6.85 ± 0.34^ax^	7.42 ± 0.10^y^	7.14 ± 0.28^A^	0.0249^ψ^	0.1399	0.2946
FSBM2.5	7.63 ± 0.19^b^	7.61 ± 0.05	7.62 ± 0.01^B^			
FSBM5	7.63 ± 0.13^b^	7.75 ± 0.11	7.69 ± 0.06^B^			
Mean	7.37 ± 0.26	7.59 ± 0.09				
Day 36						
CON	8.47 ± 0.32^a^	8.60 ± 0.12	8.54 ± 0.07^A^	0.0200^ψ^	0.5381	0.6849
FSBM2.5	9.31 ± 0.25^bc^	9.17 ± 0.10	9.25 ± 0.07^B^			
FSBM5	9.20 ± 0.24^ab^	8.95 ± 0.05	9.07 ± 0.12^B^			
Mean	8.99 ± 0.27	8.91 ± 0.17				
^abc/xy^ Means with different superscript within a column (abc) or row (xy) differ significantly. ^ABC/XY^ Means with different superscript within a column (ABC) or row (XY) differ significantly.						
^ψ^ Significant effects of supplementation level feed group (S), bird weight (B) or their interaction (S*B)

**Table 7:** Means (± SEM) for bird initial hatch weight and supplementation level of fermented soybean meal on lactic acid bacterial count from cecal contents of chick raised to 39 days.

Cecal lactic acid bacterial count (log CFU/g)						
Supplementation level	Bird initial hatch weight			P-value		
	High	Low	Mean	S	B	S*B
Day 20						
CON	8.53 ± 0.12^a^	8.52 ± 0.15^a^	8.53 ± 0.01^A^	< 0.001^ψ^	0.0143^ψ^	0.3413
FSBM2.5	9.33 ± 0.07^b^	9.05 ± 0.13^bc^	9.19 ± 0.14^BC^			
FSBM5	9.14 ± 0.08^by^	8.80 ± 0.07^abx^	8.97 ± 0.17^AB^			
Mean	9.00 ± 0.24^Y^	8.79 ± 0.15^X^				
Day 36						
CON	8.19 ± 0.25	8.15 ± 0.22	8.17 ± 0.03^A^	0.0037^ψ^	0.7648	0.8688
FSBM2.5	8.84 ± 0.17	8.92 ± 0.14	8.88 ± 0.04^B^			
FSBM5	8.56 ± 0.23	8.70 ± 0.15	8.63 ± 0.07^B^			
Mean	8.53 ± 0.18	8.59 ± 0.23				
^abc/xy^ Means with different superscript within a column (abc) or row (xy) differ significantly. ^ABC/XY^ Means with different superscript within a column (ABC) or row (XY) differ significantly.						
^ψ^ Significant effects of supplementation level feed group (S), bird weight (B) or their interaction (S*B)						

The microbiological analysis of both fecal and cecal contents reveals that FSBM serves as an effective prebiotic-like substrate, consistently promoting the proliferation of beneficial LAB throughout the gastrointestinal tract of broilers. The synchronized increase in LAB counts—observed from day 10 through 36 in the feces and mirrored in the cecal contents at days 20 and 36—underscores the systemic impact of fermented feeds on the gut ecosystem. These findings are consistent with previous studies, which reported that birds fed diets where soybean meal was supplemented with FSBM exhibited significantly higher cecal LAB counts at days 10 and 24 (Jazi et al., 2019). Furthermore, replacing 25% and 50% of SBM with FSBM has been shown to significantly increase the relative abundance of beneficial genera, including *Lachnospiraceae, Lachnoclostridium*, and *Lactobacillus*, by day 36 (Li et al., 2020). Fermentation not only reduces anti-nutritional factors but also produces organic acids and bioactive peptides that create a favorable acidic environment (Predescu et al., 2024). This environment selectively facilitates the growth of acid-tolerant beneficial bacteria, such as *Lactobacillus* spp., while potentially inhibiting pathogenic species through competitive exclusion (Sugiharto and Ranjitkar, 2019). The consistently higher LAB counts in the FSBM-supplemented groups, particularly at the 2.5% inclusion level, correlate strongly with the improved FCR and ADG observed during the grower phase and the overall experimental period. The comparable, and occasionally superior, LAB counts in the FSBM2.5 group relative to FSBM5 suggests a plateau effect, where 2.5% supplementation provides sufficient prebiotic substrates to saturate available niches for beneficial bacteria without the potential metabolic overhead or altered passage rates associated with higher inclusion levels. A robust LAB population in the cecum, a primary site for microbial fermentation, is associated with the reduction in luminal pH observed in this study. This acidic environment is likely attributed to the microbial production of short-chain fatty acids (SCFAs), which not only suppress pathogens but also enhance nutrient absorption by strengthening the mucosal immune barrier (Predescu et al., 2024; Sugiharto and Ranjitkar, 2019). This biological synergy explains why birds in the FSBM2.5 group could extract and assimilate nutrients more effectively from a smaller volume of feed, as evidenced by their superior feed efficiency. Regarding the influence of initial hatch weight, the dynamics of LAB colonization exhibited a noteworthy temporal shift. The initial disparity observed at day 1—where low weight birds displayed higher fecal LAB levels—could potentially be linked to variations in yolk sac absorption rates (Şahan et al., 2014), differences in early environmental exposure, the stochastic nature of early microbial colonization in neonates (Stamilla et al., 2021), and pecking behavior immediately after hatching (Maki et al., 2019). However, by day 20, high weight birds showed a higher LAB density in both fecal and cecal samples, likely driven by their higher feed intake and greater gastrointestinal capacity during the peak grower phase (Tona et al., 2004). Interestingly, this hatch weight-related disparity disappeared by the end of the study (day 36), where microbial profiles converged across all bird weights. This microbial convergence indicates that FSBM acts as a stabilizing agent that accelerates the maturation of the gut ecosystem in lower weight individuals, effectively narrowing the developmental gap and supporting the observed growth recovery pattern toward the end of the production cycle. By maintaining a high density of beneficial microbes through the finisher phase, FSBM supplementation effectively provided lower weight individuals with the digestive efficiency and microbial stability needed to accelerate growth and align with the standard growth trajectory.

In parallel with these microbial shifts, the pH values of cecal contents were significantly influenced by the dietary treatments (Table 8). While the inclusion of FSBM did not significantly alter the pH of the trial diets—with values remaining relatively stable from 5.50 ± 0.026 to 5.66 ± 0.025—a notable reduction in cecal pH was observed starting from day 20. In both initial hatch weight categories, birds fed 2.5% and 5% FSBM exhibited significantly lower cecal pH values compared to the control group. This trend persisted through day 36, where the cecal pH remained significantly reduced in the FSBM groups for both high and low weight birds. Notably, among the low weight birds at day 36, the FSBM2.5 group achieved the lowest pH value (6.41 ± 0.13), while the control group maintained the highest pH at 6.97 ± 0.09.

**Table 8:** Average (Mean ± SEM) pH value of trial feed and cecal content.

Bird initial hatch weight	Experimental group	pH values^a^		
		Feed	Cecal content	
			Day 20	Day 36
High	CON	5.66 ± 0.025^a^	6.70 ± 0.055^b^	6.95 ± 0.067^b^
	FSBM2.5	5.59 ± 0.017^a^	6.36 ± 0.064^a^	6.71 ± 0.046^a^
	FSBM5	5.51 ± 0.049^a^	6.46 ± 0.086^a^	6.79 ± 0.079^a^
	P-value	> 0.05	0.0066	0.04
Low	CON	5.59 ± 0.012^a^	6.87 ± 0.025^b^	6.97 ± 0.090^b^
	FSBM2.5	5.59 ± 0.032^a^	6.31 ± 0.156^a^	6.41 ± 0.131^a^
	FSBM5	5.50 ± 0.026^a^	6.42 ± 0.139^a^	6.52 ± 0.067^a^
	P-value	> 0.05	< 0.0475	< 0.0152

a Means with different superscript within a column (ab) differ significantly.

The significant reduction in cecal pH following FSBM supplementation indicates a meaningful shift in the fermentation profile and microbial activity within the hindgut. This aligns with the findings of Jazi et al. (2019) and Soumeh et al. (2019), who reported that replacing soybean meal with FSBM resulted in lower pH values across the gastrointestinal tract (crop, ileum, and ceca) of broilers at various growth stages. Similarly, Supriya et al. (2022) observed lower ileal pH values in broilers supplemented with FSBM at week 6. Since the dietary pH itself was not significantly altered by the addition of FSBM, the marked decrease in cecal pH suggests that the fermented substrate promotes the production of acid upon reaching the cecum. As demonstrated in previous results, FSBM increases the population of LAB. These bacteria ferment the pre-digested proteins and carbohydrates in FSBM to produce SCFAs and organic acids, thereby lowering the luminal pH (Sugiharto and Ranjitkar, 2019; Predescu et al., 2024). A more acidic environment (pH < 6.5) is biologically significant as it inhibits the proliferation of acid-sensitive pathogenic bacteria, such as *Salmonella* and *Clostridium perfringens* (Engberg et al., 2009; Niba et al., 2009). The stabilization of a lower cecal pH through day 36 is particularly beneficial for gut health, as a lower pH increases the solubility and absorption of essential minerals and maintains the integrity of the intestinal mucosa (Ao et al., 2009; Katu et al., 2025). This acidic environment likely contributes to the improved FCR and ADG observed in the FSBM2.5 groups. The fact that low weight birds showed the most pronounced reduction in pH by day 36 further explains their strong compensatory growth performance, as the acidified gut environment maximizes digestive efficiency during the finisher phase (Tona et al., 2004; Ao et al., 2009). This finding aligns seamlessly with the previously observed LAB counts, where the 2.5% inclusion level consistently fostered a superior microbial environment. The synchronization between the highest LAB density (prebiotic effect) and the lowest cecal pH (probiotic-like effect) in the FSBM2.5 group reinforces this dosage as the most effective and cost-efficient concentration for optimizing gut health, supporting the overall health, and ensuring flock uniformity even under challenging production conditions.

### Fecal score

Table 9 summarizes the fecal consistency scores of broiler chickens from day 1 to 10, categorized by initial hatch weight and experimental group. On the first day of the trial, all groups exhibited elevated fecal moisture levels, characterized by moderately wet droppings. Low weight birds in the control group showed the most pronounced moisture levels, presenting as severely wet excreta. From day 2 to 5, fecal consistency scores improved across all groups, generally stabilizing at a slightly wet appearance. However, birds supplemented with 5% FSBM (both high and low) maintained a slightly more fluid consistency compared to the control and FSBM2.5 groups during days 3 through 5. By the end of the 10-day period (day 6 to 10), fecal appearance further improved toward normal, well-formed dropping or a slightly wet consistency. Notably, high weight birds in the control group showed the most consistent return to normal, firm stool by day 8. Overall, both FSBM groups maintained a fecal appearance ranging from normal to slightly wet, indicating that the fecal consistency remained within a healthy and manageable range throughout the starter period.

**Table 9:** Summary of fecal score from day 1 to 10 (D1-D10) in each experimental group.

Bird initial hatch weight	Experimental group	Fecal score^a^									
		D1	D2	D3	D4	D5	D6	D7	D8	D9	D10
High	CON	3	1	1	1	1	0	1	0	0	0
	FSBM2.5	3	1	1	1	1	1	0	1	1	1
	FSBM5	3	1	2	2	2	1	0	0	1	1
Low	CON	4	1	1	1	1	1	1	1	1	1
	FSBM2.5	3	1	1	1	1	1	1	1	1	1
	FSBM5	3	2	2	2	1	0	0	1	0	1

a Score 0 = Normal; 1 = Slightly wet; 2 = Wet; 3 = Moderately wet; 4 = Severely wet.

The assessment of fecal scores provides a practical indicator of gastrointestinal health and the bird’s adaptation to the experimental diets. The moderately to severely wet droppings observed on day 1 are characteristic of the physiological stress and dietary transition typical of newly hatched chicks (Noy and Sklan, 1997; Dibner et al., 1998; Uni and Ferket, 2004). Interestingly, the higher moisture content in the low weight bird control group compared to the FSBM-supplemented low weight birds may suggest that FSBM provides an initial stabilizing effect on the neonatal gut. The occasional increase in fecal moisture in the FSBM5 group between days 3 and 5 can be attributed to the inherent characteristics of fermented ingredients. Fermentation increase the dietary organic acid load (Predescu et al., 2024), which can exert a mild osmotic effect in the hindgut, drawing additional water into the feces (Etheridge et al., 1984). However, these scores remained within a manageable range and stabilized rapidly, indicating efficient digestive systems adaptation to the 5% inclusion rate. Normalizing fecal moisture is critical for preventing “wet litter” conditions and associated welfare issue, such as footpad dermatitis (Dunlop et al., 2016; Taira et al., 2014). Overall, while the 5% inclusion level caused a transient increase in fecal moisture during the first week, both FSBM levels supported a stable gut environment on day 10. The fecal score data suggests that broiler chickens adapt well to FSBM supplementation, supporting its status as a safe and effective feed additive. Furthermore, the consistency observed in the FSBM2.5 group aligns with the growth performance (ADG and FCR) results reported elsewhere in this study.

### Hematological and biochemical parameters

The hematological and biochemical parameters of chicken serum on day 20 are summarized in Table 10. Overall measured values across the experimental groups (control, FSBM2.5, and FSBM5) for both high and low weight birds remained within the established physiological reference ranges for broiler chickens. RBC counts, HGB concentrations, and HCT levels were consistent across all treatments, falling within normal physiological limits. Similarly, WBC counts and the differential leukocyte profiles (heterophils, lymphocytes, monocytes, eosinophils, and basophils) showed no abnormal shifts, indicating the absence of systemic infection or inflammatory responses. Liver function indicators, specifically AST and ALT, remained within normal ranges, with AST levels notably lower in the FSBM-supplemented groups than in the control. Kidney function, assessed via BUN, was also within healthy limits. Furthermore, serum protein profiles (total protein, albumin, and globulin) and lipid profiles (triglyceride and cholesterol) showed no significant deviations, suggesting an absence of metabolic distress or nutritional deficiency.

**Table 10:** Hematological and biochemical parameters of chicken serum collected on day 20 of experiment.

Item	Unit	High initial weight			Low initial weight			Normal range^a^
		CON	FSBM2.5	FSBM5	CON	FSBM2.5	FSBM5	
RBC	× 10^6^/ul	2.16 ± 0.16	2.26 ± 0.26	2.52 ± 0.07	2.13 ± 0.27	2.33 ± 0.08	2.04 ± 0.30	2.0–2.8
HGB	g/dL	7.95 ± 0.60	7.92 ± 0.82	9.08 ± 0.189	7.22 ± 0.87	8.08 ± 0.36	7.53 ± 0.40	7.0–10.5
HCT	%	22.33 ± 1.72	23.67 ± 2.65	26.83 ± 0.97	21.97 ± 2.82	24.23 ± 1.029	23.68 ± 1.47	23.0–30.0
MCV	fL	86.23 ± 16.96	105.03 ± 1.34	106.23 ± 1.62	103.03 ± 1.02	103.87± 1.95	107.27 ± 2.10	90.0–140.0
MCH	pg	36.92 ± 0.71	35.47 ± 0.97	32.73 ± 3.27	34.22 ± 0.70	34.63 ± 0.64	34.25 ± 0.87	33.0–47.0
MCHC	g/dL	35.62 ± 0.65	33.62 ± 0.86	33.95 ± 0.56	33.22 ± 0.64	33.43 ± 0.47	31.93 ± 0.46	26.0–35.0
Thrombocyte	× 10^3^/ul	24.00 ± 1.63	26.67 ± 2.74	25.33 ± 2.80	23.33 ± 1.69	25.33 ± 3.12	23.83 ± 2.30	20.0–50.0
WBC	× 10^3^/ul	14.43 ± 1.13	14.35 ± 0.94	15.68 ± 1.11	14.88 ± 0.42	14.93 ± 0.86	14.73 ± 0.85	12.0–30.0
Heterophil	%	22.33 ± 4.21	24.83 ± 4.60	27.67 ± 3.11	23.83 ± 2.30	31.16 ± 6.47	27.17 ± 2.73	20.0–40.0
Lymphocyte	%	73.50 ± 4.24	81.17 ± 4.36	78.00 ± 3.13	80.33 ± 1.98	61.83 ± 5.64	78.67 ± 3.13	50.0–80.0
Monocyte	%	2.83 ± 0.54	2.33 ± 0.76	3.33 ± 0.67	4.00 ± 0.86	4.67 ± 1.23	2.17 ± 0.70	1.0–5.0
Eosinophil	%	0.67 ± 0.49	0.67 ± 0.42	0.17 ± 0.17	0.67 ± 0.49	1.00 ± 0.52	0.67 ± 0.49	1.0–3.0
Basophil	%	0.67 ± 0.33	1.33 ± 0.56	0.83 ± 0.31	1.17 ± 0.31	0.83 ± 0.40	1.50 ± 0.56	1.0–4.0
AST	IU/L	342.27 ± 54.01	246.60 ± 12.02	235.02 ± 13.63	250.86 ± 21.97	265.62 ± 19.25	278.17 ± 32.89	150.0–350.0
ALT	IU/L	2.85 ± 0.18	2.72 ± 0.19	2.27 ± 0.25	3.05 ± 0.29	2.57 ± 0.22	3.25 ± 0.37	5.0–15.0
CK	× 10^3^ IU/L	6.52 ± 1.69	4.79 ± 0.43	4.50 ± 0.74	5.16 ± 1.03	4.11 ± 0.61	5.51 ± 1.09	0.5–7.0
BUN	mg/dL	1.52 ± 0.18	1.07 ± 0.11	1.15 ± 0.25	1.28 ± 0.19	1.02 ± 0.23	1.27 ± 0.31	0.4-4.7
Total protein	g/dL	3.60 ± 0.10	3.45± 0.10	3.42 ± 0.15	3.50 ± 0.18	3.60 ± 0.10	3.38 ± 0.13	3.0–5.0
Albumin	g/dL	1.97 ± 0.06	1.88 ± 0.04	1.90 ± 0.08	1.90 ± 0.07	1.95 ± 0.07	1.87 ± 0.06	1.2–2.8
Globulin	g/dL	1.63 ± 0.05	1.57 ± 0.07	1.52 ± 0.08	1.60 ± 0.12	1.65 ± 0.06	1.52 ± 0.07	1.5–3.0
Triglyceride	mg/dL	87.85 ± 3.24	76.58 ± 12.34	84.95 ± 16.50	75.33 ± 9.63	93.77 ± 13.12	93.12 ± 23.25	50.0–150.0
Cholesterol	mg/dL	146.93 ± 5.11	146.67 ± 4.32	138.15 ± 8.62	120.03 ± 5.40	137.20 ± 8.05	122.35 ± 4.09	100.0–200.0

a The normal range values in this table were modified from previously reported data (Kaneko, et al., 2008; Thrall, et al., 2022; Weiss and Wardrop, 2011).

The primary objective of analyzing hematological and biochemical parameters is to assess the physiological health status and ensure the safety of FSBM as a dietary supplement. A critical finding of this study is that FSBM supplementation (2.5% and 5.0%) produced no adverse effects on the systemic health of the broiler chickens. The observation that all hematological parameters, particularly RBC and HGB, remained within standard reference ranges indicates that the fermentation process effectively neutralized ANFs such as phytates and tannins. This may prevented mineral sequestration and significantly enhanced nutrient bioavailability, specifically improving iron absorption, a critical factor for hemoglobin synthesis and hematopoiesis. Furthermore, the enrichment of folate and vitamin B12 during fermentation likely supported proper red blood cell maturation. Collectively, the fermented plant-based diet can successfully maintain systemic iron homeostasis and support optimal erythropoiesis without interference from inhibitory compounds (Knez et al., 2023). Our results align with previous studies report that chickens supplemented with FSBM exhibited RBC and HGB levels comparable to those of the control group (Vaisakh et al., 2019; Khonyoung et al., 2026). However, this contrasts with the findings of Chachaj et al. (2019), who reported that FSBM-supplemented birds exhibited elevated RBC and HGB levels. Furthermore, the stable WBC and differential counts confirm that the fermented substrate does not trigger an adverse immune or inflammatory response (SaeidiFard et al., 2020). These findings are in concordance with previous studies (Chachaj et al., 2019; Vaisakh et al., 2019), with the exception of the study by (Khonyoung et al., 2026), who reported that chicks supplemented with 25% FSBM exhibited higher lymphocyte levels. While elevated lymphocytes may suggest active immunomodulation, the stable counts in this study indicate that the fermented diet maintained optimal immune homeostasis without physiological stress. This suggests that systemic immunity and gut-associated lymphoid tissue (GALT) were adequately supported at a baseline level, ensuring health without excessive leukocyte mobilization (SaeidiFard et al., 2020). The concentrations of AST and ALT are sensitive biomarkers of hepatocyte integrity. In this study, these enzymes remained well within the normal ranges; notably, the FSBM-supplemented groups exhibited AST levels remained well within the normal physiological range across all groups. Since AST is a sensitive indicator of cellular damage in the liver and muscle tissues, these stable levels indicate that FSBM does not induce hepatic stress. This highlights that the pre-digestion of proteins and reduction of anti-nutritional factors during fermentation provide a safe nutrient source that efficiently maintains normal liver homeostasis. This can be attributed to the synergistic impact of metabolic relief—where the pre-digestion of proteins into bioactive small peptides and the elimination of ANFs significantly minimizes the liver’s metabolic workload. Furthermore, the enhanced antioxidant capacity associated with FSBM helps mitigate oxidative stress and preserve hepatocyte integrity, thereby preventing the leakage of enzymes into the bloodstream (Mohd Yusof et al., 2013; Lambo et al., 2024). Our findings relate to the prior results of Khonyoung et al. (2026), who reported a decrease in AST levels in birds fed with 25% and 50% FSBM. In contrast, Chachaj et al. (2019) found that birds supplemented with FSBM (10 g/kg feed) exhibited increased AST levels. Despite these fluctuations, AST levels remained stable in the study by Vaisakh et al. (2019), even at a 30% inclusion level. In addition to the liver, the kidneys are critical organs that should be monitored. Similar to the AST results, BUN levels remained low and stable in our findings, confirming that the inclusion of FSBM does not place undue metabolic stress on the kidneys. The stability of BUN serves as a strong indicator of the high protein quality within the FSBM used in this study. Typically, BUN levels rise when an animal consumes low-quality protein or excessive protein amounts, leading to increased deamination in the liver and subsequent renal excretion (Donsbough et al., 2010). This suggests that the amino acid profile of the fermented diet was well-balanced and efficiently utilized by the birds. This contrasts with the creatinine levels reported by Chachaj et al. (2019), who found that supplementation with 10% FSBM led to an elevation in creatinine, suggesting a potential negative impact on renal function. These discrepancies of hepatic and renal biomarkers across studies may be attributed to several factors, including the concentration of bioactive compounds generated during fermentation, the baseline health status of the birds, and the specific duration of the supplementation period. The stability of serum proteins and lipid profiles further supports the safety of FSBM. Our results showed that total protein, albumin, and globulin levels were within the normal physiological range. This is consistent with the findings of Chachaj et al. (2019) and Tang et al. (2012), who reported no significant differences in total protein and albumin levels between chickens fed with and without FSBM. In contrast, Vaisakh et al. (2019) observed higher levels of total protein, albumin, and globulin in chickens supplemented with FSBM by day 42. Since serum total protein and albumin levels are direct indicators of body protein anabolism and reflect the efficiency of protein deposition in tissues (Stahly et al., 1994), our findings suggest that FSBM supplementation provides an adequate and balanced supply of amino acids for protein synthesis. The maintenance of normal globulin levels further indicates a healthy immune status, suggesting that FSBM does not disrupt the local immune balance in the gut (Tsai et al., 2021). This stability may be attributed to the bioactive components in FSBM, such as antioxidants and phytochemicals, which enhance the animal’s antioxidative status, immune function and ultimately overall health (Sembratowicz et al., 2020; Obianwuna et al., 2024). Complementing the stable protein metabolism, the lipid profiles also indicated that the maintenance of normal triglyceride and cholesterol levels—consistent with observations in birds receiving FSBM in previous studies where no significant differences were observed compared to non-supplemented groups (Chachaj et al., 2019; Vaisakh et al., 2019)—demonstrates that FSBM supplementation effectively regulates lipid metabolism (Saleh et al., 2019). The stability of these lipid profiles, concurrent with optimal growth performance, suggests that the fermentation process enhances lipid digestion and absorption within the gastrointestinal tract (Mulalapele et al., 2026). Furthermore, this suggests that the dietary inclusion of FSBM provides a balanced nutrient supply that does not impose an unnecessary burden on the liver for compensatory cholesterol synthesis (Ng’onga et al., 2025). These hematological and biochemical profiles provide strong evidence for the safety of FSBM in broiler diets. The absence of adverse markers pertaining to toxicity, organ damage, or metabolic imbalance confirms that FSBM, at inclusion levels up to 5%, is a physiologically safe feed ingredient that maintains internal homeostasis while supporting optimal growth performance.

A limitation of the current study is that the experimental diets were not strictly balanced for calcium. Dietary calcium levels inadvertently increased from 1.23% in the control diet to 1.30% and 1.37% in the 2.5% and 5% FSBM diets, respectively, due to variations in calcium carbonate inclusion. While variations in dietary calcium can influence mineral metabolism and broiler performance, the optimal growth and feed efficiency observed at the intermediate 2.5% inclusion level suggests that the primary driver of the observed improvements was the functional properties of the fermented soybean meal rather than the calcium gradient. Future studies should ensure strictly isocalcic formulations to isolate the effects of FSBM completely.

## Conclusion

The supplementation with 2.5% FSBM is an effective and safe strategy for optimizing broiler production in tropical climates. By supporting physiological homeostasis and enabling compensatory growth in low initial weight chicks, this inclusion rate serves as a practical intervention to improve overall flock uniformity and productivity. Furthermore, stable hematochemical parameters confirm that FSBM is physiologically safe, supporting enhanced productivity and intestinal health without adverse systemic effects. The maintenance of normal AST and ALT levels indicates that FSBM inclusion supports normal hepatic function and does not induce metabolic stress on the liver. Ultimately, a 2.5% FSBM inclusion rate serves as a robust intervention to promote growth efficiency and maintain internal homeostasis in broilers.

## Author contribution

**Substantial Contributions:** Pawiya Pupa, Theerawit Poeikhampha, and Savek Kiatsomphob contributed substantially to the conception, design, visualization, and project administration of the work, as well as the acquisition and analysis of data. Chonticha Phumtrairat contributed substantially to the conception of the study and technical product specification. Chaiyakorn Khamson and Sombat Prasongsook contributed substantially to the investigation and curation of study data.

**Drafting or Reviewing:** Pawiya Pupa, Theerawit Poeikhampha, and Savek Kiatsomphob drafted the original manuscript. Chaiyakorn Khamson, Chonticha Phumtrairat and Sombat Prasongsook critically reviewed the manuscript for crucial intellectual content.

## Final approval

All authors have reviewed and explicitly approved the exact, final version of the manuscript prior to this submission.

## Accountability

All authors agree to be personally and publicly accountable for all aspects of the work.

## Disclosures

Chonticha Phumtrairat is an employee of Evershining Ingredient Co., Ltd., which supplied the product evaluated in this study and contributed to the study as a scientific collaborator, as described in the Author Contributions section. The remaining authors declare no financial or personal conflicts of interest.
